# Supplementary figures and images for: An autoinhibitory clamp of actin assembly constrains and directs synaptic endocytosis
Source: eLife. 2021 Jul 29;10:e69597. doi: 10.7554/eLife.69597 (PMC8321554; doi:10.7554/eLife.69597)

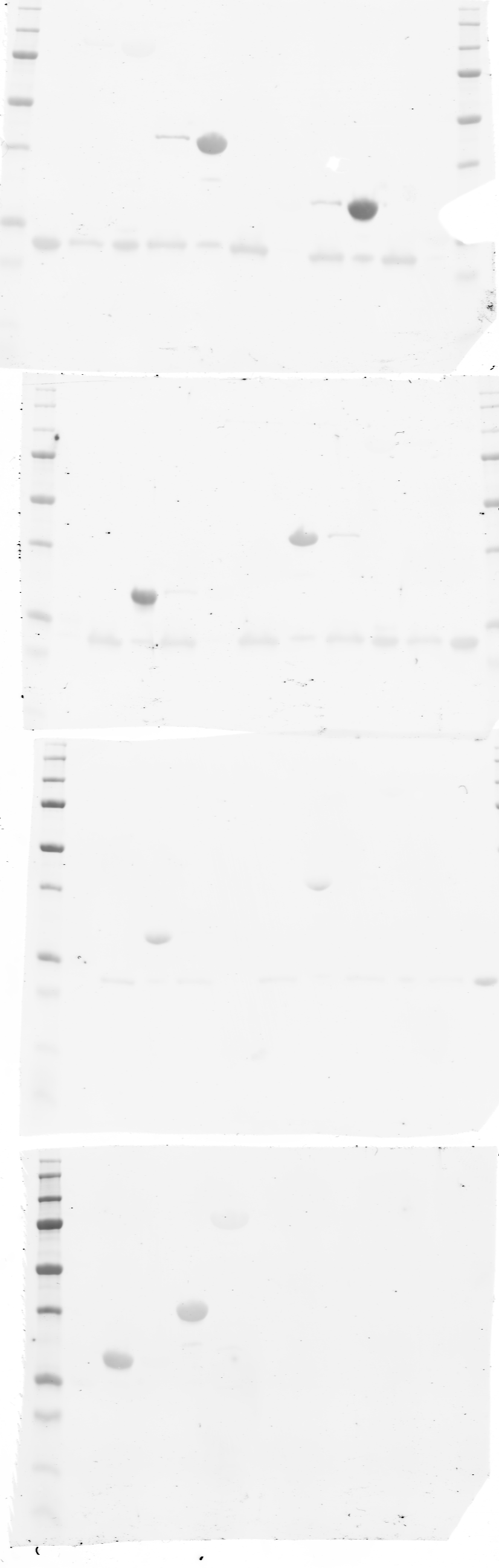

Supplement: Figure 3—source data 1. — Whole Coomassie gels measuring the interaction between GST-Dap160 fragments and Nwk proteins, as indicated in each file name. Collected and annotated gels for Figure 3B with lanes and constructs labeled. Quantification of blots for Figure 3B. Quantification of Nwk intensity in Dap160 rescue larvae, also containing raw data for Figure 3—figure supplement 2B (Dap160 transgene abundance). Source data quantifying Nwk-Dap160 transgene colocalization and intensity in Dap160 rescue larvae; also containing raw data for Figure 3—figure supplement 2C (Dap160 transgene abundance). Collected and annotated gels measuring Dap160 fragment pulldowns of Nwk SH3b for Figure 3—figure supplement 1A with lanes and constructs labeled. Raw gels for Figure 3—figure supplement 1A. Raw data quantifying Figure 3—figure supplement 1A. Collected and annotated gels measuring Dap160 fragment pulldowns of Nwk FBAR in Figure 3—figure supplement 1B with lanes and constructs labeled. Raw gels for Figure 3—figure supplement 1B. Data quantifying Dap160 fragment pulldowns of Nwk FBAR. Raw data quantifying Dap160 abundance in control and dap160 rnai-expressing NMJs. [file elife-69597-fig3-data1.zip › Figure 3 - source data 1/Figure 3-supplement 1A_raw gels_DapPulldownOfSH3b.tif]

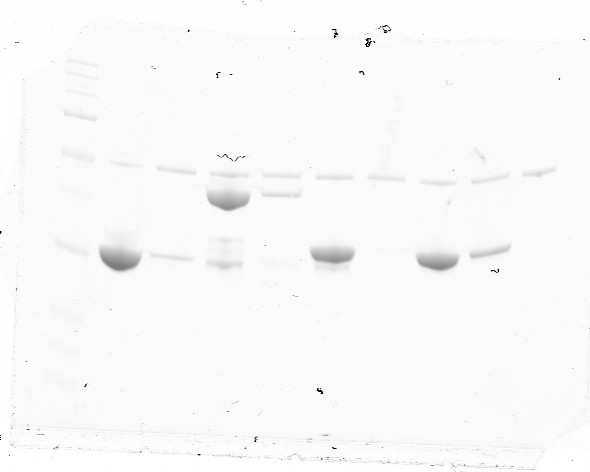

Supplement: Figure 3—source data 1. — Whole Coomassie gels measuring the interaction between GST-Dap160 fragments and Nwk proteins, as indicated in each file name. Collected and annotated gels for Figure 3B with lanes and constructs labeled. Quantification of blots for Figure 3B. Quantification of Nwk intensity in Dap160 rescue larvae, also containing raw data for Figure 3—figure supplement 2B (Dap160 transgene abundance). Source data quantifying Nwk-Dap160 transgene colocalization and intensity in Dap160 rescue larvae; also containing raw data for Figure 3—figure supplement 2C (Dap160 transgene abundance). Collected and annotated gels measuring Dap160 fragment pulldowns of Nwk SH3b for Figure 3—figure supplement 1A with lanes and constructs labeled. Raw gels for Figure 3—figure supplement 1A. Raw data quantifying Figure 3—figure supplement 1A. Collected and annotated gels measuring Dap160 fragment pulldowns of Nwk FBAR in Figure 3—figure supplement 1B with lanes and constructs labeled. Raw gels for Figure 3—figure supplement 1B. Data quantifying Dap160 fragment pulldowns of Nwk FBAR. Raw data quantifying Dap160 abundance in control and dap160 rnai-expressing NMJs. [file elife-69597-fig3-data1.zip › Figure 3 - source data 1/Figure 3-supplement 1B_raw gel_Gel1.TIF]

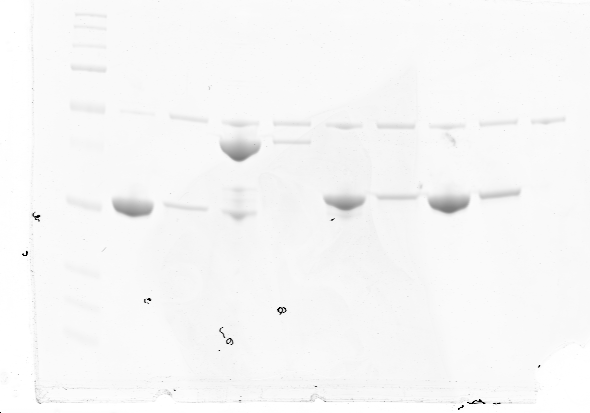

Supplement: Figure 3—source data 1. — Whole Coomassie gels measuring the interaction between GST-Dap160 fragments and Nwk proteins, as indicated in each file name. Collected and annotated gels for Figure 3B with lanes and constructs labeled. Quantification of blots for Figure 3B. Quantification of Nwk intensity in Dap160 rescue larvae, also containing raw data for Figure 3—figure supplement 2B (Dap160 transgene abundance). Source data quantifying Nwk-Dap160 transgene colocalization and intensity in Dap160 rescue larvae; also containing raw data for Figure 3—figure supplement 2C (Dap160 transgene abundance). Collected and annotated gels measuring Dap160 fragment pulldowns of Nwk SH3b for Figure 3—figure supplement 1A with lanes and constructs labeled. Raw gels for Figure 3—figure supplement 1A. Raw data quantifying Figure 3—figure supplement 1A. Collected and annotated gels measuring Dap160 fragment pulldowns of Nwk FBAR in Figure 3—figure supplement 1B with lanes and constructs labeled. Raw gels for Figure 3—figure supplement 1B. Data quantifying Dap160 fragment pulldowns of Nwk FBAR. Raw data quantifying Dap160 abundance in control and dap160 rnai-expressing NMJs. [file elife-69597-fig3-data1.zip › Figure 3 - source data 1/FIgure 3-supplement 1B_raw gel_gel2.tif]

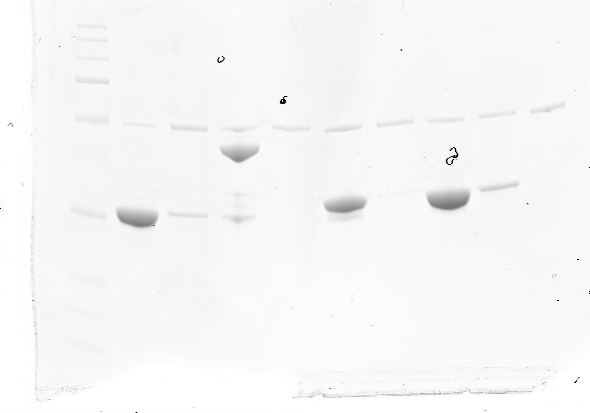

Supplement: Figure 3—source data 1. — Whole Coomassie gels measuring the interaction between GST-Dap160 fragments and Nwk proteins, as indicated in each file name. Collected and annotated gels for Figure 3B with lanes and constructs labeled. Quantification of blots for Figure 3B. Quantification of Nwk intensity in Dap160 rescue larvae, also containing raw data for Figure 3—figure supplement 2B (Dap160 transgene abundance). Source data quantifying Nwk-Dap160 transgene colocalization and intensity in Dap160 rescue larvae; also containing raw data for Figure 3—figure supplement 2C (Dap160 transgene abundance). Collected and annotated gels measuring Dap160 fragment pulldowns of Nwk SH3b for Figure 3—figure supplement 1A with lanes and constructs labeled. Raw gels for Figure 3—figure supplement 1A. Raw data quantifying Figure 3—figure supplement 1A. Collected and annotated gels measuring Dap160 fragment pulldowns of Nwk FBAR in Figure 3—figure supplement 1B with lanes and constructs labeled. Raw gels for Figure 3—figure supplement 1B. Data quantifying Dap160 fragment pulldowns of Nwk FBAR. Raw data quantifying Dap160 abundance in control and dap160 rnai-expressing NMJs. [file elife-69597-fig3-data1.zip › Figure 3 - source data 1/FIgure 3-supplement 1B_raw gel_gel3.tif]

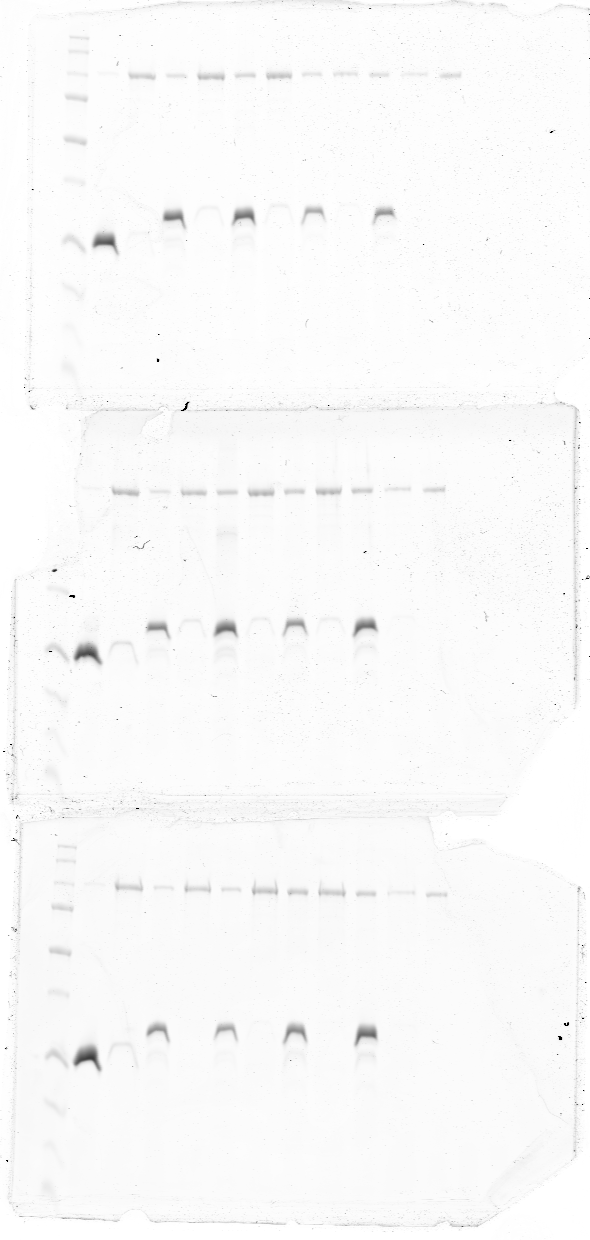

Supplement: Figure 3—source data 1. — Whole Coomassie gels measuring the interaction between GST-Dap160 fragments and Nwk proteins, as indicated in each file name. Collected and annotated gels for Figure 3B with lanes and constructs labeled. Quantification of blots for Figure 3B. Quantification of Nwk intensity in Dap160 rescue larvae, also containing raw data for Figure 3—figure supplement 2B (Dap160 transgene abundance). Source data quantifying Nwk-Dap160 transgene colocalization and intensity in Dap160 rescue larvae; also containing raw data for Figure 3—figure supplement 2C (Dap160 transgene abundance). Collected and annotated gels measuring Dap160 fragment pulldowns of Nwk SH3b for Figure 3—figure supplement 1A with lanes and constructs labeled. Raw gels for Figure 3—figure supplement 1A. Raw data quantifying Figure 3—figure supplement 1A. Collected and annotated gels measuring Dap160 fragment pulldowns of Nwk FBAR in Figure 3—figure supplement 1B with lanes and constructs labeled. Raw gels for Figure 3—figure supplement 1B. Data quantifying Dap160 fragment pulldowns of Nwk FBAR. Raw data quantifying Dap160 abundance in control and dap160 rnai-expressing NMJs. [file elife-69597-fig3-data1.zip › Figure 3 - source data 1/Figure 3B_raw gel_Dap160C.TIF]

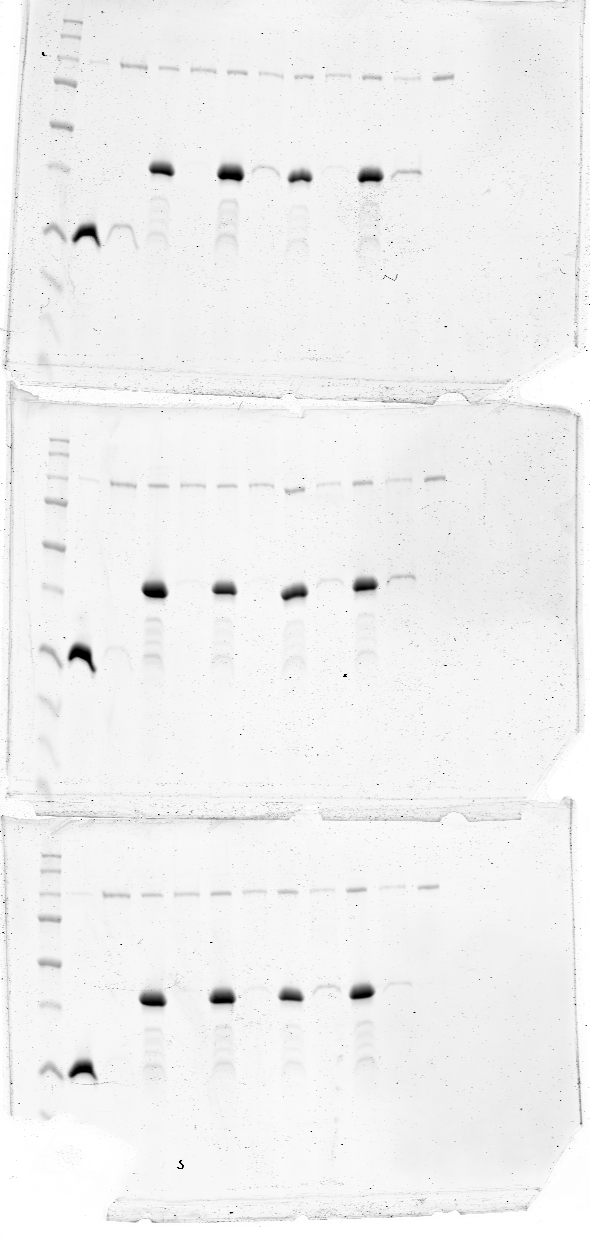

Supplement: Figure 3—source data 1. — Whole Coomassie gels measuring the interaction between GST-Dap160 fragments and Nwk proteins, as indicated in each file name. Collected and annotated gels for Figure 3B with lanes and constructs labeled. Quantification of blots for Figure 3B. Quantification of Nwk intensity in Dap160 rescue larvae, also containing raw data for Figure 3—figure supplement 2B (Dap160 transgene abundance). Source data quantifying Nwk-Dap160 transgene colocalization and intensity in Dap160 rescue larvae; also containing raw data for Figure 3—figure supplement 2C (Dap160 transgene abundance). Collected and annotated gels measuring Dap160 fragment pulldowns of Nwk SH3b for Figure 3—figure supplement 1A with lanes and constructs labeled. Raw gels for Figure 3—figure supplement 1A. Raw data quantifying Figure 3—figure supplement 1A. Collected and annotated gels measuring Dap160 fragment pulldowns of Nwk FBAR in Figure 3—figure supplement 1B with lanes and constructs labeled. Raw gels for Figure 3—figure supplement 1B. Data quantifying Dap160 fragment pulldowns of Nwk FBAR. Raw data quantifying Dap160 abundance in control and dap160 rnai-expressing NMJs. [file elife-69597-fig3-data1.zip › Figure 3 - source data 1/Figure 3B_raw gel_Dap160CD.TIF]

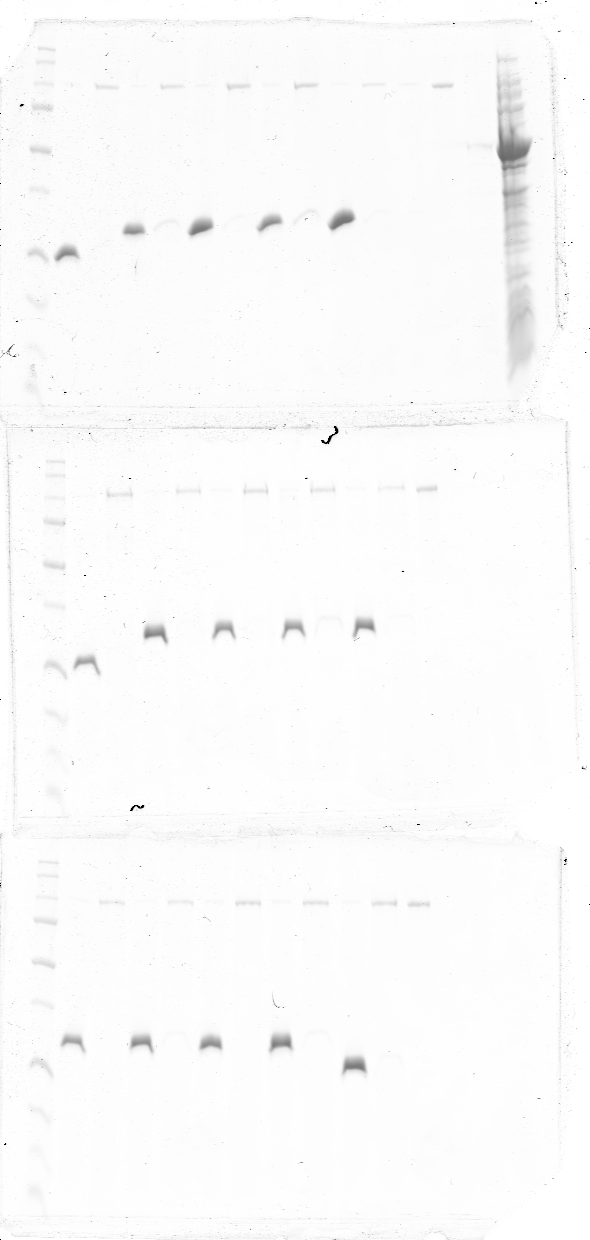

Supplement: Figure 3—source data 1. — Whole Coomassie gels measuring the interaction between GST-Dap160 fragments and Nwk proteins, as indicated in each file name. Collected and annotated gels for Figure 3B with lanes and constructs labeled. Quantification of blots for Figure 3B. Quantification of Nwk intensity in Dap160 rescue larvae, also containing raw data for Figure 3—figure supplement 2B (Dap160 transgene abundance). Source data quantifying Nwk-Dap160 transgene colocalization and intensity in Dap160 rescue larvae; also containing raw data for Figure 3—figure supplement 2C (Dap160 transgene abundance). Collected and annotated gels measuring Dap160 fragment pulldowns of Nwk SH3b for Figure 3—figure supplement 1A with lanes and constructs labeled. Raw gels for Figure 3—figure supplement 1A. Raw data quantifying Figure 3—figure supplement 1A. Collected and annotated gels measuring Dap160 fragment pulldowns of Nwk FBAR in Figure 3—figure supplement 1B with lanes and constructs labeled. Raw gels for Figure 3—figure supplement 1B. Data quantifying Dap160 fragment pulldowns of Nwk FBAR. Raw data quantifying Dap160 abundance in control and dap160 rnai-expressing NMJs. [file elife-69597-fig3-data1.zip › Figure 3 - source data 1/Figure 3B_raw gel_Dap160D.TIF]

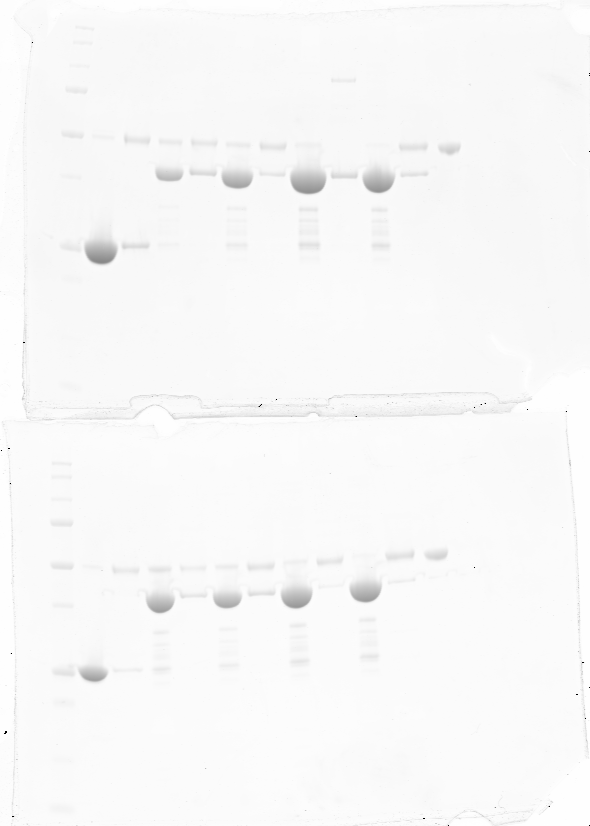

Supplement: Figure 3—source data 1. — Whole Coomassie gels measuring the interaction between GST-Dap160 fragments and Nwk proteins, as indicated in each file name. Collected and annotated gels for Figure 3B with lanes and constructs labeled. Quantification of blots for Figure 3B. Quantification of Nwk intensity in Dap160 rescue larvae, also containing raw data for Figure 3—figure supplement 2B (Dap160 transgene abundance). Source data quantifying Nwk-Dap160 transgene colocalization and intensity in Dap160 rescue larvae; also containing raw data for Figure 3—figure supplement 2C (Dap160 transgene abundance). Collected and annotated gels measuring Dap160 fragment pulldowns of Nwk SH3b for Figure 3—figure supplement 1A with lanes and constructs labeled. Raw gels for Figure 3—figure supplement 1A. Raw data quantifying Figure 3—figure supplement 1A. Collected and annotated gels measuring Dap160 fragment pulldowns of Nwk FBAR in Figure 3—figure supplement 1B with lanes and constructs labeled. Raw gels for Figure 3—figure supplement 1B. Data quantifying Dap160 fragment pulldowns of Nwk FBAR. Raw data quantifying Dap160 abundance in control and dap160 rnai-expressing NMJs. [file elife-69597-fig3-data1.zip › Figure 3 - source data 1/Figure 3B_raw gel_DapCD-NwkG_1and3.TIF]

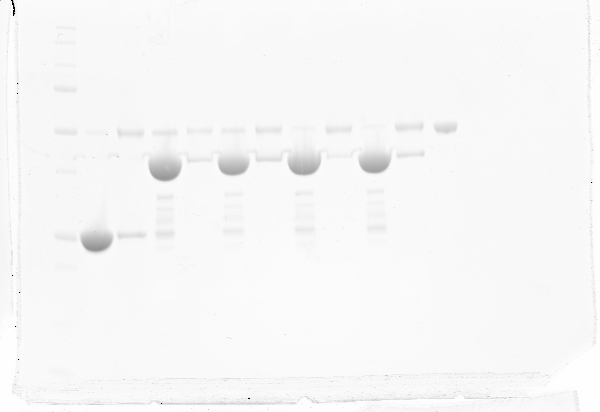

Supplement: Figure 3—source data 1. — Whole Coomassie gels measuring the interaction between GST-Dap160 fragments and Nwk proteins, as indicated in each file name. Collected and annotated gels for Figure 3B with lanes and constructs labeled. Quantification of blots for Figure 3B. Quantification of Nwk intensity in Dap160 rescue larvae, also containing raw data for Figure 3—figure supplement 2B (Dap160 transgene abundance). Source data quantifying Nwk-Dap160 transgene colocalization and intensity in Dap160 rescue larvae; also containing raw data for Figure 3—figure supplement 2C (Dap160 transgene abundance). Collected and annotated gels measuring Dap160 fragment pulldowns of Nwk SH3b for Figure 3—figure supplement 1A with lanes and constructs labeled. Raw gels for Figure 3—figure supplement 1A. Raw data quantifying Figure 3—figure supplement 1A. Collected and annotated gels measuring Dap160 fragment pulldowns of Nwk FBAR in Figure 3—figure supplement 1B with lanes and constructs labeled. Raw gels for Figure 3—figure supplement 1B. Data quantifying Dap160 fragment pulldowns of Nwk FBAR. Raw data quantifying Dap160 abundance in control and dap160 rnai-expressing NMJs. [file elife-69597-fig3-data1.zip › Figure 3 - source data 1/Figure 3B_raw gel_DapCD-NwkG_Gel2.TIF]

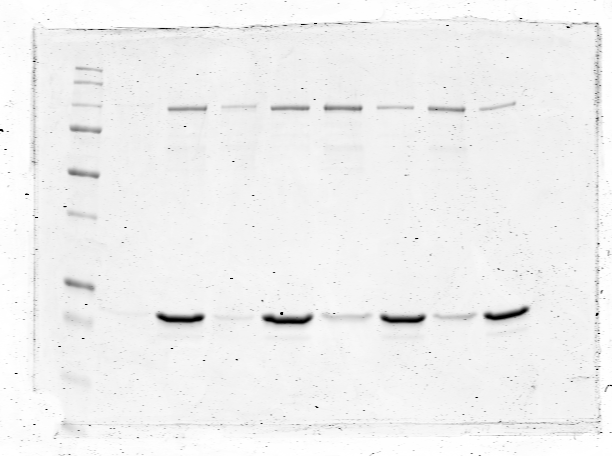

Supplement: Figure 5—source data 1. — Source data quantifying liposome cosedimentation of Nwk with increasing concentrations of Dap160SH3CD. Annotated gels quantifying liposome cosedimentation of Nwk with increasing concentrations of Dap160SH3CD. Raw gels quantifying liposome cosedimentation of Nwk with increasing concentrations of Dap160SH3CD. Annotated gels quantifying liposome cosedimentation of Nwk and Dap160 fragments with increasing concentrations of PI(4,5)P2. Raw gels quantifying liposome cosedimentation of Nwk and Dap160 fragments with increasing concentrations of PI(4,5)P2. Source data quantifying liposome cosedimentation of Nwk and Dap160 fragments with increasing concentrations of PI(4,5)P2. Source data quantifying FRAP recovery and curve fitting of Nwk::GFP in control and dap160 RNAi-expressing neuromuscular junctions (NMJs). Annotated gels quantifying liposome cosedimentation of NwkΔSH3b and Dap160 fragments. Raw gels quantifying liposome cosedimentation of NwkΔSH3b and Dap160 fragments. Source data quantifying liposome cosedimentation of NwkΔSH3b and Dap160 fragments. [file elife-69597-fig5-data1.zip › Figure 5 - source data 1/Figure 5A_raw gel_His CD A.TIF]

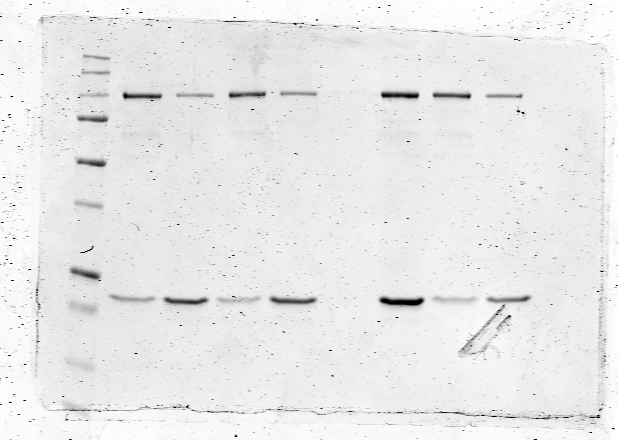

Supplement: Figure 5—source data 1. — Source data quantifying liposome cosedimentation of Nwk with increasing concentrations of Dap160SH3CD. Annotated gels quantifying liposome cosedimentation of Nwk with increasing concentrations of Dap160SH3CD. Raw gels quantifying liposome cosedimentation of Nwk with increasing concentrations of Dap160SH3CD. Annotated gels quantifying liposome cosedimentation of Nwk and Dap160 fragments with increasing concentrations of PI(4,5)P2. Raw gels quantifying liposome cosedimentation of Nwk and Dap160 fragments with increasing concentrations of PI(4,5)P2. Source data quantifying liposome cosedimentation of Nwk and Dap160 fragments with increasing concentrations of PI(4,5)P2. Source data quantifying FRAP recovery and curve fitting of Nwk::GFP in control and dap160 RNAi-expressing neuromuscular junctions (NMJs). Annotated gels quantifying liposome cosedimentation of NwkΔSH3b and Dap160 fragments. Raw gels quantifying liposome cosedimentation of NwkΔSH3b and Dap160 fragments. Source data quantifying liposome cosedimentation of NwkΔSH3b and Dap160 fragments. [file elife-69597-fig5-data1.zip › Figure 5 - source data 1/Figure 5A_raw gel_His CD B.TIF]

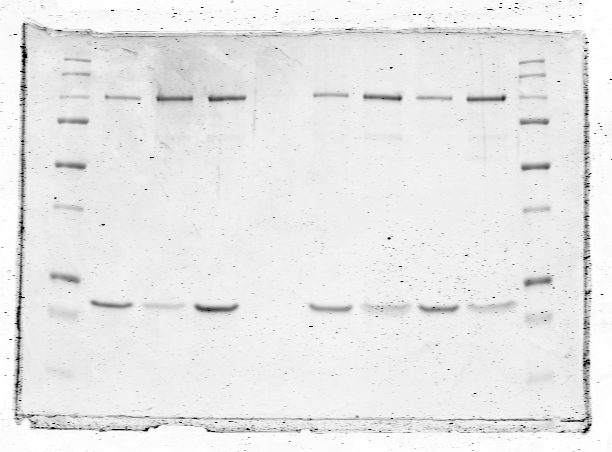

Supplement: Figure 5—source data 1. — Source data quantifying liposome cosedimentation of Nwk with increasing concentrations of Dap160SH3CD. Annotated gels quantifying liposome cosedimentation of Nwk with increasing concentrations of Dap160SH3CD. Raw gels quantifying liposome cosedimentation of Nwk with increasing concentrations of Dap160SH3CD. Annotated gels quantifying liposome cosedimentation of Nwk and Dap160 fragments with increasing concentrations of PI(4,5)P2. Raw gels quantifying liposome cosedimentation of Nwk and Dap160 fragments with increasing concentrations of PI(4,5)P2. Source data quantifying liposome cosedimentation of Nwk and Dap160 fragments with increasing concentrations of PI(4,5)P2. Source data quantifying FRAP recovery and curve fitting of Nwk::GFP in control and dap160 RNAi-expressing neuromuscular junctions (NMJs). Annotated gels quantifying liposome cosedimentation of NwkΔSH3b and Dap160 fragments. Raw gels quantifying liposome cosedimentation of NwkΔSH3b and Dap160 fragments. Source data quantifying liposome cosedimentation of NwkΔSH3b and Dap160 fragments. [file elife-69597-fig5-data1.zip › Figure 5 - source data 1/Figure 5A_raw gel_His CD C.TIF]

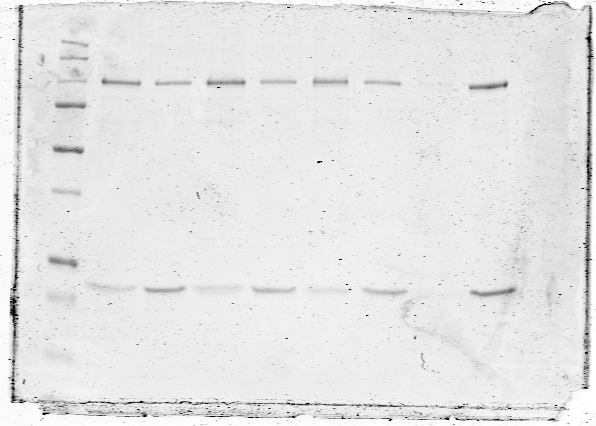

Supplement: Figure 5—source data 1. — Source data quantifying liposome cosedimentation of Nwk with increasing concentrations of Dap160SH3CD. Annotated gels quantifying liposome cosedimentation of Nwk with increasing concentrations of Dap160SH3CD. Raw gels quantifying liposome cosedimentation of Nwk with increasing concentrations of Dap160SH3CD. Annotated gels quantifying liposome cosedimentation of Nwk and Dap160 fragments with increasing concentrations of PI(4,5)P2. Raw gels quantifying liposome cosedimentation of Nwk and Dap160 fragments with increasing concentrations of PI(4,5)P2. Source data quantifying liposome cosedimentation of Nwk and Dap160 fragments with increasing concentrations of PI(4,5)P2. Source data quantifying FRAP recovery and curve fitting of Nwk::GFP in control and dap160 RNAi-expressing neuromuscular junctions (NMJs). Annotated gels quantifying liposome cosedimentation of NwkΔSH3b and Dap160 fragments. Raw gels quantifying liposome cosedimentation of NwkΔSH3b and Dap160 fragments. Source data quantifying liposome cosedimentation of NwkΔSH3b and Dap160 fragments. [file elife-69597-fig5-data1.zip › Figure 5 - source data 1/Figure 5A_raw gel_His CD D.TIF]

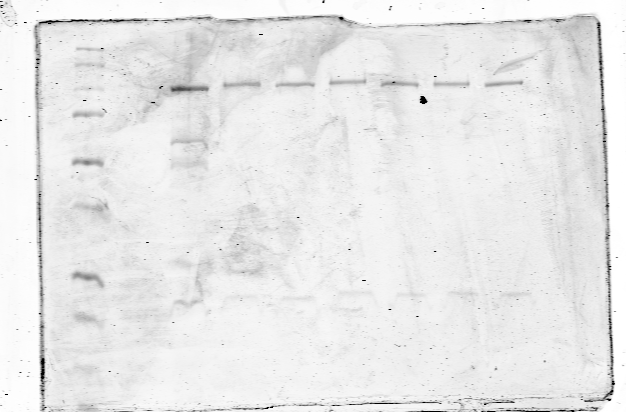

Supplement: Figure 5—source data 1. — Source data quantifying liposome cosedimentation of Nwk with increasing concentrations of Dap160SH3CD. Annotated gels quantifying liposome cosedimentation of Nwk with increasing concentrations of Dap160SH3CD. Raw gels quantifying liposome cosedimentation of Nwk with increasing concentrations of Dap160SH3CD. Annotated gels quantifying liposome cosedimentation of Nwk and Dap160 fragments with increasing concentrations of PI(4,5)P2. Raw gels quantifying liposome cosedimentation of Nwk and Dap160 fragments with increasing concentrations of PI(4,5)P2. Source data quantifying liposome cosedimentation of Nwk and Dap160 fragments with increasing concentrations of PI(4,5)P2. Source data quantifying FRAP recovery and curve fitting of Nwk::GFP in control and dap160 RNAi-expressing neuromuscular junctions (NMJs). Annotated gels quantifying liposome cosedimentation of NwkΔSH3b and Dap160 fragments. Raw gels quantifying liposome cosedimentation of NwkΔSH3b and Dap160 fragments. Source data quantifying liposome cosedimentation of NwkΔSH3b and Dap160 fragments. [file elife-69597-fig5-data1.zip › Figure 5 - source data 1/Figure 5A_raw gel_His CD E.TIF]

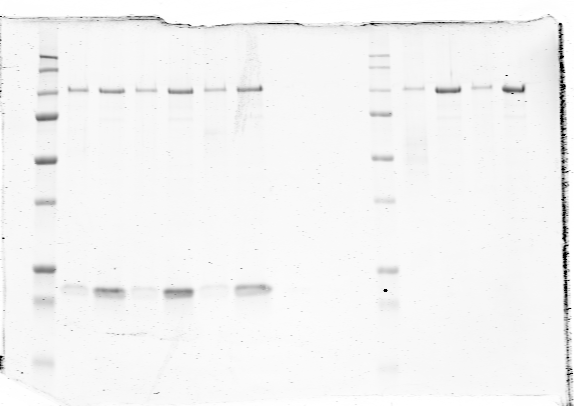

Supplement: Figure 5—source data 1. — Source data quantifying liposome cosedimentation of Nwk with increasing concentrations of Dap160SH3CD. Annotated gels quantifying liposome cosedimentation of Nwk with increasing concentrations of Dap160SH3CD. Raw gels quantifying liposome cosedimentation of Nwk with increasing concentrations of Dap160SH3CD. Annotated gels quantifying liposome cosedimentation of Nwk and Dap160 fragments with increasing concentrations of PI(4,5)P2. Raw gels quantifying liposome cosedimentation of Nwk and Dap160 fragments with increasing concentrations of PI(4,5)P2. Source data quantifying liposome cosedimentation of Nwk and Dap160 fragments with increasing concentrations of PI(4,5)P2. Source data quantifying FRAP recovery and curve fitting of Nwk::GFP in control and dap160 RNAi-expressing neuromuscular junctions (NMJs). Annotated gels quantifying liposome cosedimentation of NwkΔSH3b and Dap160 fragments. Raw gels quantifying liposome cosedimentation of NwkΔSH3b and Dap160 fragments. Source data quantifying liposome cosedimentation of NwkΔSH3b and Dap160 fragments. [file elife-69597-fig5-data1.zip › Figure 5 - source data 1/Figure 5B_raw gel_Dap160CDandC.tif]

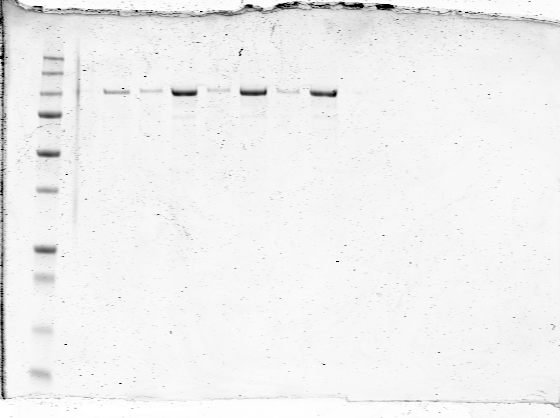

Supplement: Figure 5—source data 1. — Source data quantifying liposome cosedimentation of Nwk with increasing concentrations of Dap160SH3CD. Annotated gels quantifying liposome cosedimentation of Nwk with increasing concentrations of Dap160SH3CD. Raw gels quantifying liposome cosedimentation of Nwk with increasing concentrations of Dap160SH3CD. Annotated gels quantifying liposome cosedimentation of Nwk and Dap160 fragments with increasing concentrations of PI(4,5)P2. Raw gels quantifying liposome cosedimentation of Nwk and Dap160 fragments with increasing concentrations of PI(4,5)P2. Source data quantifying liposome cosedimentation of Nwk and Dap160 fragments with increasing concentrations of PI(4,5)P2. Source data quantifying FRAP recovery and curve fitting of Nwk::GFP in control and dap160 RNAi-expressing neuromuscular junctions (NMJs). Annotated gels quantifying liposome cosedimentation of NwkΔSH3b and Dap160 fragments. Raw gels quantifying liposome cosedimentation of NwkΔSH3b and Dap160 fragments. Source data quantifying liposome cosedimentation of NwkΔSH3b and Dap160 fragments. [file elife-69597-fig5-data1.zip › Figure 5 - source data 1/Figure 5B_raw gel_NwkB2.tif]

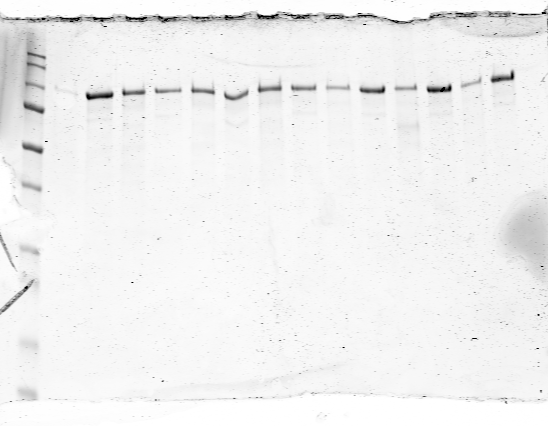

Supplement: Figure 5—source data 1. — Source data quantifying liposome cosedimentation of Nwk with increasing concentrations of Dap160SH3CD. Annotated gels quantifying liposome cosedimentation of Nwk with increasing concentrations of Dap160SH3CD. Raw gels quantifying liposome cosedimentation of Nwk with increasing concentrations of Dap160SH3CD. Annotated gels quantifying liposome cosedimentation of Nwk and Dap160 fragments with increasing concentrations of PI(4,5)P2. Raw gels quantifying liposome cosedimentation of Nwk and Dap160 fragments with increasing concentrations of PI(4,5)P2. Source data quantifying liposome cosedimentation of Nwk and Dap160 fragments with increasing concentrations of PI(4,5)P2. Source data quantifying FRAP recovery and curve fitting of Nwk::GFP in control and dap160 RNAi-expressing neuromuscular junctions (NMJs). Annotated gels quantifying liposome cosedimentation of NwkΔSH3b and Dap160 fragments. Raw gels quantifying liposome cosedimentation of NwkΔSH3b and Dap160 fragments. Source data quantifying liposome cosedimentation of NwkΔSH3b and Dap160 fragments. [file elife-69597-fig5-data1.zip › Figure 5 - source data 1/Figure 5B_raw gel_NwkB_Dap160C.tif]

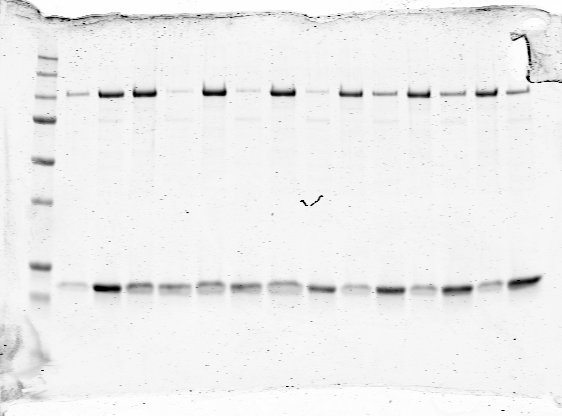

Supplement: Figure 5—source data 1. — Source data quantifying liposome cosedimentation of Nwk with increasing concentrations of Dap160SH3CD. Annotated gels quantifying liposome cosedimentation of Nwk with increasing concentrations of Dap160SH3CD. Raw gels quantifying liposome cosedimentation of Nwk with increasing concentrations of Dap160SH3CD. Annotated gels quantifying liposome cosedimentation of Nwk and Dap160 fragments with increasing concentrations of PI(4,5)P2. Raw gels quantifying liposome cosedimentation of Nwk and Dap160 fragments with increasing concentrations of PI(4,5)P2. Source data quantifying liposome cosedimentation of Nwk and Dap160 fragments with increasing concentrations of PI(4,5)P2. Source data quantifying FRAP recovery and curve fitting of Nwk::GFP in control and dap160 RNAi-expressing neuromuscular junctions (NMJs). Annotated gels quantifying liposome cosedimentation of NwkΔSH3b and Dap160 fragments. Raw gels quantifying liposome cosedimentation of NwkΔSH3b and Dap160 fragments. Source data quantifying liposome cosedimentation of NwkΔSH3b and Dap160 fragments. [file elife-69597-fig5-data1.zip › Figure 5 - source data 1/Figure 5B_raw gel_NwkB_Dap160CD.tif]

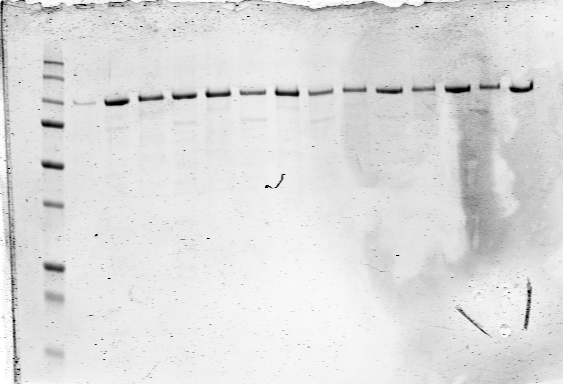

Supplement: Figure 5—source data 1. — Source data quantifying liposome cosedimentation of Nwk with increasing concentrations of Dap160SH3CD. Annotated gels quantifying liposome cosedimentation of Nwk with increasing concentrations of Dap160SH3CD. Raw gels quantifying liposome cosedimentation of Nwk with increasing concentrations of Dap160SH3CD. Annotated gels quantifying liposome cosedimentation of Nwk and Dap160 fragments with increasing concentrations of PI(4,5)P2. Raw gels quantifying liposome cosedimentation of Nwk and Dap160 fragments with increasing concentrations of PI(4,5)P2. Source data quantifying liposome cosedimentation of Nwk and Dap160 fragments with increasing concentrations of PI(4,5)P2. Source data quantifying FRAP recovery and curve fitting of Nwk::GFP in control and dap160 RNAi-expressing neuromuscular junctions (NMJs). Annotated gels quantifying liposome cosedimentation of NwkΔSH3b and Dap160 fragments. Raw gels quantifying liposome cosedimentation of NwkΔSH3b and Dap160 fragments. Source data quantifying liposome cosedimentation of NwkΔSH3b and Dap160 fragments. [file elife-69597-fig5-data1.zip › Figure 5 - source data 1/Figure 5B_raw gel_NwkB_Dap160C_17436.tif]

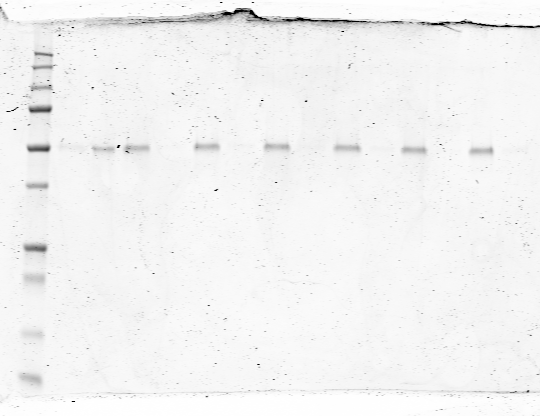

Supplement: Figure 5—source data 1. — Source data quantifying liposome cosedimentation of Nwk with increasing concentrations of Dap160SH3CD. Annotated gels quantifying liposome cosedimentation of Nwk with increasing concentrations of Dap160SH3CD. Raw gels quantifying liposome cosedimentation of Nwk with increasing concentrations of Dap160SH3CD. Annotated gels quantifying liposome cosedimentation of Nwk and Dap160 fragments with increasing concentrations of PI(4,5)P2. Raw gels quantifying liposome cosedimentation of Nwk and Dap160 fragments with increasing concentrations of PI(4,5)P2. Source data quantifying liposome cosedimentation of Nwk and Dap160 fragments with increasing concentrations of PI(4,5)P2. Source data quantifying FRAP recovery and curve fitting of Nwk::GFP in control and dap160 RNAi-expressing neuromuscular junctions (NMJs). Annotated gels quantifying liposome cosedimentation of NwkΔSH3b and Dap160 fragments. Raw gels quantifying liposome cosedimentation of NwkΔSH3b and Dap160 fragments. Source data quantifying liposome cosedimentation of NwkΔSH3b and Dap160 fragments. [file elife-69597-fig5-data1.zip › Figure 5 - source data 1/Figure 5B_raw gel_NwkG.tif]

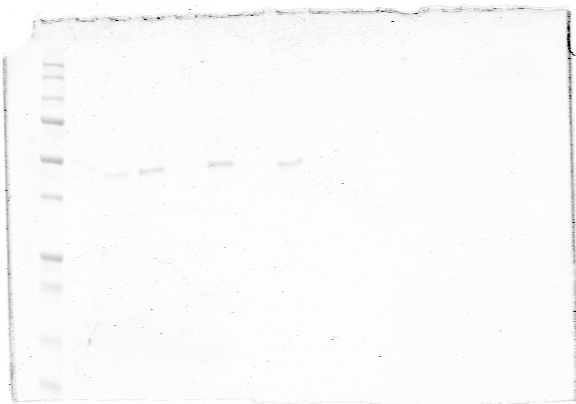

Supplement: Figure 5—source data 1. — Source data quantifying liposome cosedimentation of Nwk with increasing concentrations of Dap160SH3CD. Annotated gels quantifying liposome cosedimentation of Nwk with increasing concentrations of Dap160SH3CD. Raw gels quantifying liposome cosedimentation of Nwk with increasing concentrations of Dap160SH3CD. Annotated gels quantifying liposome cosedimentation of Nwk and Dap160 fragments with increasing concentrations of PI(4,5)P2. Raw gels quantifying liposome cosedimentation of Nwk and Dap160 fragments with increasing concentrations of PI(4,5)P2. Source data quantifying liposome cosedimentation of Nwk and Dap160 fragments with increasing concentrations of PI(4,5)P2. Source data quantifying FRAP recovery and curve fitting of Nwk::GFP in control and dap160 RNAi-expressing neuromuscular junctions (NMJs). Annotated gels quantifying liposome cosedimentation of NwkΔSH3b and Dap160 fragments. Raw gels quantifying liposome cosedimentation of NwkΔSH3b and Dap160 fragments. Source data quantifying liposome cosedimentation of NwkΔSH3b and Dap160 fragments. [file elife-69597-fig5-data1.zip › Figure 5 - source data 1/Figure 5B_raw gel_NwkG2.5.tif]

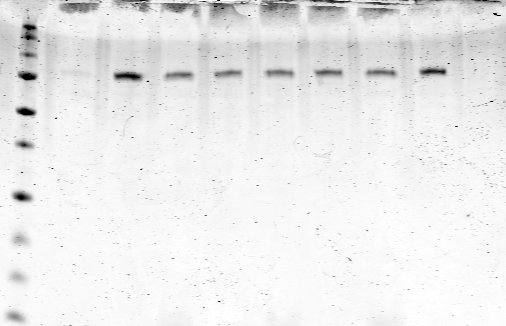

Supplement: Figure 5—source data 1. — Source data quantifying liposome cosedimentation of Nwk with increasing concentrations of Dap160SH3CD. Annotated gels quantifying liposome cosedimentation of Nwk with increasing concentrations of Dap160SH3CD. Raw gels quantifying liposome cosedimentation of Nwk with increasing concentrations of Dap160SH3CD. Annotated gels quantifying liposome cosedimentation of Nwk and Dap160 fragments with increasing concentrations of PI(4,5)P2. Raw gels quantifying liposome cosedimentation of Nwk and Dap160 fragments with increasing concentrations of PI(4,5)P2. Source data quantifying liposome cosedimentation of Nwk and Dap160 fragments with increasing concentrations of PI(4,5)P2. Source data quantifying FRAP recovery and curve fitting of Nwk::GFP in control and dap160 RNAi-expressing neuromuscular junctions (NMJs). Annotated gels quantifying liposome cosedimentation of NwkΔSH3b and Dap160 fragments. Raw gels quantifying liposome cosedimentation of NwkΔSH3b and Dap160 fragments. Source data quantifying liposome cosedimentation of NwkΔSH3b and Dap160 fragments. [file elife-69597-fig5-data1.zip › Figure 5 - source data 1/Figure 5S1A_raw gel_NwkC.tif]

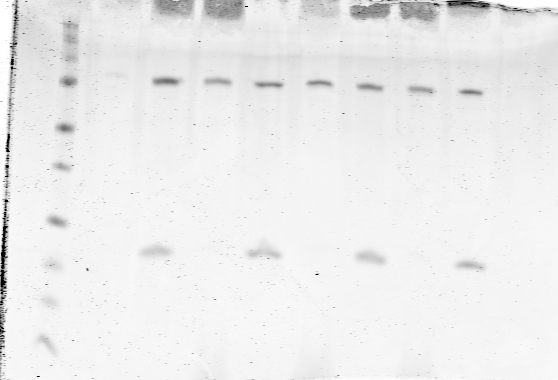

Supplement: Figure 5—source data 1. — Source data quantifying liposome cosedimentation of Nwk with increasing concentrations of Dap160SH3CD. Annotated gels quantifying liposome cosedimentation of Nwk with increasing concentrations of Dap160SH3CD. Raw gels quantifying liposome cosedimentation of Nwk with increasing concentrations of Dap160SH3CD. Annotated gels quantifying liposome cosedimentation of Nwk and Dap160 fragments with increasing concentrations of PI(4,5)P2. Raw gels quantifying liposome cosedimentation of Nwk and Dap160 fragments with increasing concentrations of PI(4,5)P2. Source data quantifying liposome cosedimentation of Nwk and Dap160 fragments with increasing concentrations of PI(4,5)P2. Source data quantifying FRAP recovery and curve fitting of Nwk::GFP in control and dap160 RNAi-expressing neuromuscular junctions (NMJs). Annotated gels quantifying liposome cosedimentation of NwkΔSH3b and Dap160 fragments. Raw gels quantifying liposome cosedimentation of NwkΔSH3b and Dap160 fragments. Source data quantifying liposome cosedimentation of NwkΔSH3b and Dap160 fragments. [file elife-69597-fig5-data1.zip › Figure 5 - source data 1/Figure 5S1A_raw gel_NwkC_DapCD.tif]

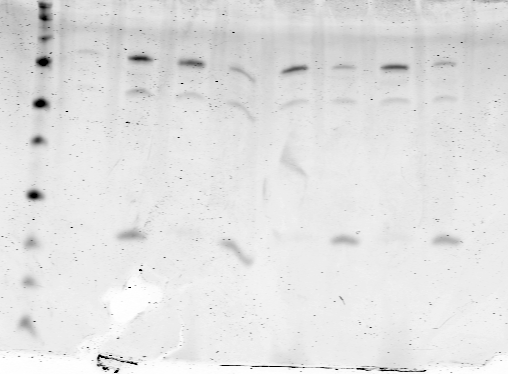

Supplement: Figure 5—source data 1. — Source data quantifying liposome cosedimentation of Nwk with increasing concentrations of Dap160SH3CD. Annotated gels quantifying liposome cosedimentation of Nwk with increasing concentrations of Dap160SH3CD. Raw gels quantifying liposome cosedimentation of Nwk with increasing concentrations of Dap160SH3CD. Annotated gels quantifying liposome cosedimentation of Nwk and Dap160 fragments with increasing concentrations of PI(4,5)P2. Raw gels quantifying liposome cosedimentation of Nwk and Dap160 fragments with increasing concentrations of PI(4,5)P2. Source data quantifying liposome cosedimentation of Nwk and Dap160 fragments with increasing concentrations of PI(4,5)P2. Source data quantifying FRAP recovery and curve fitting of Nwk::GFP in control and dap160 RNAi-expressing neuromuscular junctions (NMJs). Annotated gels quantifying liposome cosedimentation of NwkΔSH3b and Dap160 fragments. Raw gels quantifying liposome cosedimentation of NwkΔSH3b and Dap160 fragments. Source data quantifying liposome cosedimentation of NwkΔSH3b and Dap160 fragments. [file elife-69597-fig5-data1.zip › Figure 5 - source data 1/Figure 5S1A_raw gel_NwkC_Wasp_DapCD.tif]

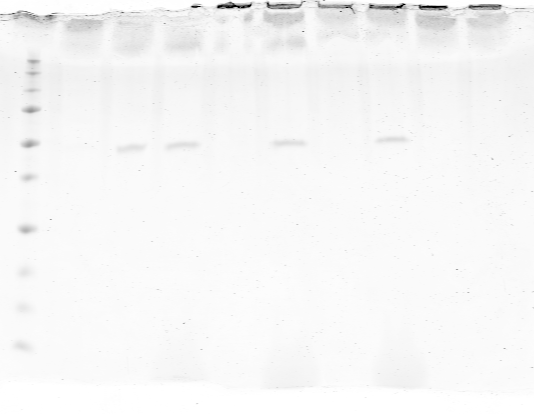

Supplement: Figure 5—source data 1. — Source data quantifying liposome cosedimentation of Nwk with increasing concentrations of Dap160SH3CD. Annotated gels quantifying liposome cosedimentation of Nwk with increasing concentrations of Dap160SH3CD. Raw gels quantifying liposome cosedimentation of Nwk with increasing concentrations of Dap160SH3CD. Annotated gels quantifying liposome cosedimentation of Nwk and Dap160 fragments with increasing concentrations of PI(4,5)P2. Raw gels quantifying liposome cosedimentation of Nwk and Dap160 fragments with increasing concentrations of PI(4,5)P2. Source data quantifying liposome cosedimentation of Nwk and Dap160 fragments with increasing concentrations of PI(4,5)P2. Source data quantifying FRAP recovery and curve fitting of Nwk::GFP in control and dap160 RNAi-expressing neuromuscular junctions (NMJs). Annotated gels quantifying liposome cosedimentation of NwkΔSH3b and Dap160 fragments. Raw gels quantifying liposome cosedimentation of NwkΔSH3b and Dap160 fragments. Source data quantifying liposome cosedimentation of NwkΔSH3b and Dap160 fragments. [file elife-69597-fig5-data1.zip › Figure 5 - source data 1/Figure 5S1A_raw gel_NwkG.tif]

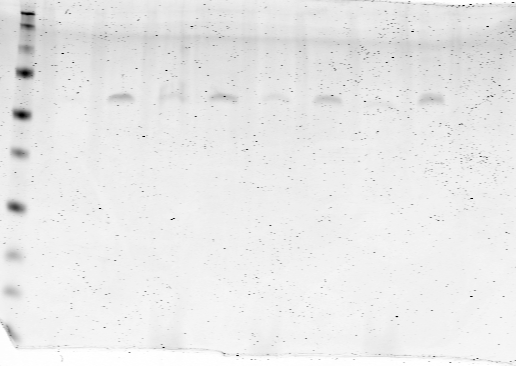

Supplement: Figure 5—source data 1. — Source data quantifying liposome cosedimentation of Nwk with increasing concentrations of Dap160SH3CD. Annotated gels quantifying liposome cosedimentation of Nwk with increasing concentrations of Dap160SH3CD. Raw gels quantifying liposome cosedimentation of Nwk with increasing concentrations of Dap160SH3CD. Annotated gels quantifying liposome cosedimentation of Nwk and Dap160 fragments with increasing concentrations of PI(4,5)P2. Raw gels quantifying liposome cosedimentation of Nwk and Dap160 fragments with increasing concentrations of PI(4,5)P2. Source data quantifying liposome cosedimentation of Nwk and Dap160 fragments with increasing concentrations of PI(4,5)P2. Source data quantifying FRAP recovery and curve fitting of Nwk::GFP in control and dap160 RNAi-expressing neuromuscular junctions (NMJs). Annotated gels quantifying liposome cosedimentation of NwkΔSH3b and Dap160 fragments. Raw gels quantifying liposome cosedimentation of NwkΔSH3b and Dap160 fragments. Source data quantifying liposome cosedimentation of NwkΔSH3b and Dap160 fragments. [file elife-69597-fig5-data1.zip › Figure 5 - source data 1/Figure 5S1A_raw gel_WASp.tif]

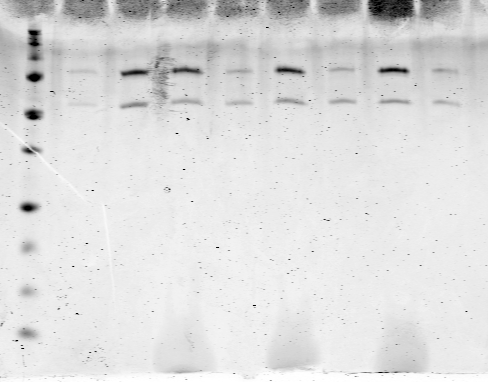

Supplement: Figure 5—source data 1. — Source data quantifying liposome cosedimentation of Nwk with increasing concentrations of Dap160SH3CD. Annotated gels quantifying liposome cosedimentation of Nwk with increasing concentrations of Dap160SH3CD. Raw gels quantifying liposome cosedimentation of Nwk with increasing concentrations of Dap160SH3CD. Annotated gels quantifying liposome cosedimentation of Nwk and Dap160 fragments with increasing concentrations of PI(4,5)P2. Raw gels quantifying liposome cosedimentation of Nwk and Dap160 fragments with increasing concentrations of PI(4,5)P2. Source data quantifying liposome cosedimentation of Nwk and Dap160 fragments with increasing concentrations of PI(4,5)P2. Source data quantifying FRAP recovery and curve fitting of Nwk::GFP in control and dap160 RNAi-expressing neuromuscular junctions (NMJs). Annotated gels quantifying liposome cosedimentation of NwkΔSH3b and Dap160 fragments. Raw gels quantifying liposome cosedimentation of NwkΔSH3b and Dap160 fragments. Source data quantifying liposome cosedimentation of NwkΔSH3b and Dap160 fragments. [file elife-69597-fig5-data1.zip › Figure 5 - source data 1/Figure 5S1A_raw gel_Wasp_NwkC.tif]
