# Supplementary material for: An autoinhibitory clamp of actin assembly constrains and directs synaptic endocytosis
Source: eLife. 2021 Jul 29;10:e69597. doi: 10.7554/eLife.69597 (PMC8321554; doi:10.7554/eLife.69597)
Supplement: Supplementary file 1. [file elife-69597-supp1.docx]

**Supplemental Table 1. Summary of genotypes and statistics for all experiments in this study.**

| **Figure/**  **Experiment** | **Genotype/Conditions** | **N** | **Statistical Test(s)** |
| --- | --- | --- | --- |
| 1A-D  GFP::Actin  GMA  Lifeact::Ruby | C155-GAL4/+ or Y; UAS-GFP::actin/+  C155-GAL4/+ or Y; UAS-GMA/+  C155-GAL4/+ or Y; UAS-lifeact::Ruby/+ | 823 patches/9 NMJ/5 larvae  819 patches/15 NMJ/6 larvae  363 patches/7 NMJ/3 larvae | NA |
| 1E-F  Arp3  BRP | C155-GAL4/UAS-Arp3::GFP; UAS-lifeact::Ruby/+  C155-GAL4/+; UAS-lifeact::Ruby/BRP::GFP^[MIMIC]^ | 12 NMJ/3 larvae  13 NMJ/3 larvae | Kruskal-Wallis + Dunn’s test  (with Fig 7C) |
| 1G-I  Control  WASp | C155-GAL4/+ or Y; UAS-GMA/+  C155-GAL4/+ or Y; wsp1,e,UAS-GMA/wsp1,e | 832 patches/18 NMJ/8 larvae  532 patches/15 NMJ/6 larvae | Kolmogorov-Smirnoff (G) Welch's t test (H) |
|  |  |  |  |
|  |  |  |  |
| 1S1A-B | C155-GAL4/+ or Y; UAS-GMA/+ | 1606 patches/20 NMJ/8 larvae | Kolmogorov-Smirnoff |
| 1S1C-E  Control  *wsp* RNAi | C155-GAL4/Y; UAS-GMA/UAS-luciferaseRNAi  C155-GAL4/Y; UAS-GMA/UAS-WASpRNAi | 709 patches/15 NMJ/6 larvae  286 patches/14 NMJ/5 larvae | Kolmogorov-Smirnoff (H)  Mann-Whitney (I)  t test (J) |
| 1S1F  Control  *wsp* RNAi | C155-GAL4/Y; UAS-myc::WASp/+; /UAS-mCherryRNAi/+  C155-GAL4/Y; UAS-myc::WASp/+; /UAS-*wsp* RNAi/+ | 18NMJ/3 larvae  24NMJ/4 larvae | t-test |
|  |  |  |  |
| 1S2 | Same experiment as Fig 1 G-I |  |  |
|  |  |  |  |
| 2A | Nwk::GFP^[MIMIC]^ |  |  |
| 2B | Vglut-GAL4/Y; CD8::RFP/+; Nwk::GFP/+ |  |  |
| 2C-D | C155-GAL4/Y; UAS-WASp::myc/+; UAS-GMA/+ | 14 NMJ/3 larvae | NA |
| 2E-F | C155-GAL4/Y; UAS-WASp::myc/+; UAS-GMA/+ | 12 NMJ/3 larvae | NA |
|  |  |  |  |
| 3B  GST-X  Nwk^1-731^ | 5μg  3μg | 3 independent mixtures per group | NA |
| 3B  GST-CD  Nwk^1-428^  Nwk^1-731^ | 1.6μM CD, 1.2μM C/D  1.5μM  0.8μM | 3 independent mixtures per group | NA |
| 3D  Dap^FL^  Dap^ΔD^  Dap^ΔCD^  Dap^RNAi^ | rescues=C155-GAL4/+; dap160^Δ1^/Df3450; X  X=UAS-Dap^FL^::mCherry/+  X=UAS-Dap^ΔD^/+  X=UAS-Dap^ΔCD^/+  C155-GAL4, UAS-Dcr2/Y; UAS Dap160-RNAi/+ | 18 NMJ/3 larvae  18 NMJ/3 larvae  17 NMJ/3 larvae  16 NMJ/3 larvae | ANOVA+Tukey’s multiple comparison test |
| 3E  Dap^FL^  Dap^ΔD^  Dap^ΔCD^ | rescues= C155-GAL4/+; dap160^Δ1^/Df3450; X  X=UAS-Dap^FL^::mCherry/+  X=UAS-Dap^ΔD^/+  X=UAS-Dap^ΔCD^/+ | 18 NMJ/3 larvae  18 NMJ/3 larvae  15 NMJ/3 larvae | Kruskal-Wallis + Dunn’s multiple comparison test |
|  |  |  |  |
|  |  |  |  |
| **Figure/**  **Experiment** | **Genotype/Conditions** | **N** | **Statistical Test(s)** |
| 3 S1A  Nwk^607-731^ | 7μM |  |  |
| 3 S1B  GST-X  Nwk^1-428^ | .375mg/mL (CD=8.5μM, C/D=11μM)  1.5μM | 3 independent mixtures per group | ANOVA+Tukey’s multiple comparison test |
|  |  |  |  |
| 3S1C | Same experiment as Figure 3B | Same experiment as Fig 3B | ANOVA+Tukey’s multiple comparison test |
|  |  |  |  |
| 3S2B | Same experiment as Figure 3D | Same experiment as Fig 3D | ANOVA+Tukey’s multiple comparison test |
| 3S2C | Same experiment as Figure 3E | Same experiment as Figure 3E | ANOVA+Tukey’s |
| 3S2D  Control  Dap160RNAi | vglut/Y; UAS-Dcr2/+; UAS-Nwk::GFP^MiMIC^/UAS-luciferaseRNAi  vglut/Y; UAS-dcr2/+; Nwk::GFP^MiMIC^/Dap160^RNAi^ | 11NMJ/3larvae  11NMJ/3larvae | t test |
|  |  |  |  |
| 4A  Actin  Arp2/3  WASp  Nwk1-731  DapCD  DapC | 2μM  50nM  50nM  500nM  2μM  2μM | (1) 2 replicates  (2) 2 replicates  (3) 3 replicates  (4) 3 replicates  (5) 5 replicates  (6) 2 replicates  Replicates are independent mixtures | ANOVA+  Tukey’s multiple comparison test |
| 4B  Actin  Arp2/3  WASp  Nwk1-731  DapCD  PI(4,5)P_2_ | 2μM  50nM  50nM  100nM  500nM  2μM 10% PI(4,5)P2 liposomes | (1) 2 replicates  (2) 2 replicates  (3) 3 replicates  (4) 3 replicates  (5) 3 replicates  Replicates are independent mixtures | ANOVA+  Tukey’s multiple comparison test |
| 4C  Nwk^1-633^  Nwk^1-731+^Dap^CD^ | 2μM OG-actin, 50nM Arp2/3, 50nM WASp  500nM Nwk^1-633^::SNAP549  500nM Nwk^1-731^::SNAP549+2μM Dap160^SH3CD^ | 41 droplets  22 droplets | NA |
|  |  |  |  |
|  |  |  |  |
| 5A  Nwk^1-428^  Nwk^1-754^  Dap^CD^ | DOPC/DOPE/DOPS/PI(4,5)P_2_ = 70/15/5/10  3μM  1.125μM  1.69-6.75μM | 3 independent mixtures per group | ANOVA+  Tukey’s multiple comparison test |
| 5B  Nwk^1-XXX^  Dap160^X^ | DOPC/DOPE/DOPS/PI(4,5)P_2_ = 80-x/15/5/x  2μM  6μM | 3 independent mixtures per group; except 2 replicates for:  2.5%PIP2-Nwk^1-428^  2.5%-Nwk^1-731^+Dap^SH3C^ | ANOVA+  Tukey’s multiple comparison test |
| 5C  Nwk^1-731^  WASp  Dap160^CD^ | DOPC/DOPE/DOPS/PI(4,5)P_2_ = 70/15/5/10  1μM  1μM  3μM | 3 independent mixtures per group | ANOVA+  Tukey’s multiple comparison test |
| **Figure/**  **Experiment** | **Genotype/Conditions** | **N** | **Statistical Test(s)** |
| 5D  Nwk^1-731^  Nwk/WASp/Dap | 5% PI(4,5)P_2_ GUVs  500nM Nwk^1-731^::SNAP549  250nM Nwk^1-731^::SNAP549, 250nM WASp, 1.25μM Dap160^SH3CD^ | Representative from:  11 GUVs imaged  12 GUVs imaged | NA |
| 5E-F  Control  Dap160RNAi | vglut/Y; UAS-Dcr2/+; UAS-Nwk::GFP^MiMIC^/UAS-luciferaseRNAi  vglut/Y; UAS-dcr2/+; Nwk::GFP^MiMIC^/Dap160^RNAi^ | 9 NMJs/4 larvae  10 NMJs/5 larvae | Two step association curve |
|  |  |  |  |
| 5S1A-B  Nwk^1-633^  WASp  Dap160^CD^ | DOPC/DOPE/DOPS/PI(4,5)P_2_ = 75/15/5/5  1µM  2 µM  3µM | 3 independent mixtures per group | ANOVA+  Tukey’s multiple comparison test |
| 5S1C  Nwk^1-731^  WASp  Dap160^CD^ | DOPC/DOPE/DOPS/PI(4,5)P_2_ = 70/15/5/10  1μM  1μM  3μM | 3 independent mixtures per group | ANOVA+  Tukey’s multiple comparison test |
| 5S1D  Nwk^1-754^  WASp  Dap160^SH3CD^ | DOPC/DOPE/DOPS/PI(4,5)P_2_ = 70/15/5/10  250nM  1μM  250nM5% PI(4,5)P_2_ GUVs | Representative from:  5 GUVs imaged  10 GUVs imaged | NA |
|  |  |  |  |
|  |  |  |  |
| 6A-C  Control  *nwk*^1/2^ | C155-GAL4/+ or Y; UAS-GMA/+  C155-GAL4/+ or Y; UAS-GMA, *nwk*^2^,*h*/ *nwk*^1^ | 1606 patches/20 NMJ/8 larvae  1928 patches/22 NMJ/8 larvae | Mann-Whitney (B)  Kolm.-Smirnoff (C) |
| 6D-F  FL  ΔCD | C155-GAL4/+; dap160^Δ1^/Df3450; UAS-GMA/X  X=UAS-Dap^FL^::mCherry/+  X=UAS-Dap^ΔCD^/+ | 1279 patches/14 NMJ/7 larvae  1937 patches/17 NMJ/7 larvae | Mann-Whitney (E)  Kolm.-Smirnoff (F) |
|  |  |  |  |
|  |  |  |  |
| 6S1A-B | Same experiment as 6A-C | As 6A-C |  |
|  |  |  |  |
| 6S2 | Same experiment as 6A-C | As 6A-C | t-test (B-C) |
|  |  |  |  |
| 7A-B  Clc::GFP  BRP::GFP | C155-GAL4/+; UAS-lifeact::Ruby/+; UAS-Clc::GFP/+  C155-GAL4/+; UAS-lifeact::Ruby/BRP::GFP^[MIMIC]^ | 9 NMJ/3 larvae  13 NMJ/3 larvae | Kruskal-Wallis + Dunn’s test  (With Fig 1E-F) |
| 7C | C155-GAL4/+; UAS-lifeact::Ruby/AP2::GFP | 14 NMJ/3 larvae | NA |
| 7D-E  Control  *shi*TS1 | C155-GAL4/Y; UAS-GMA/+  C155-GAL4, shiTS1/Y; UAS-GMA/+ | 669 patches/16 NMJ/8 larvae  901 patches/19 NMJ/8 larvae | Welch's t test (D) |
| 7F-G  Control  *nwk*^1/2^  *shi*^TS1^ | C155-GAL4/Y; UAS-GFP/+  C155-GAL4/Y; UAS-GFP/+; *nwk*^1^/*nwk*^2^*h*  C155-GAL4, shi^TS1^/Y; UAS-GFP/+ | 23 NMJ/4 larvae  24 NMJ/4 larvae  23 NMJ/4 larvae | Kruskal-Wallis + Dunn’s multiple comparison test |
| 7H-I  Dap^FL^  Dap^ΔD^  Dap^ΔCD^  *shi*^TS!^ | rescues= C155-GAL4/+; dap160^Δ1^/Df3450; X  X=UAS-Dap^FL^::mCherry/+  X=UAS-Dap^ΔD^/+  X=UAS-Dap^ΔCD^/+  C155, shiTS1/Y x UAS-RFP | 32 NMJs/8 larvae  12 NMJs/3 larvae  12 NMJs/3 larvae  8 NMJs/2 larvae | Unpaired t-tests to dish-matched controls |
| **Figure/**  **Experiment** | **Genotype/Conditions** | **N** | **Statistical Test(s)** |
| 7S1A  Clc::GFP  AP2::GFP | C155-GAL4/+; UAS-Clc::GFP/+  AP2::GFP^KI^ | 6 boutons  7 boutons | NA |
| 7S1B-C  AP2 | C155-GAL4/+; UAS-lifeact::Ruby/AP2::GFP | 14 NMJ/3 larvae | NA |
|  |  |  |  |
| Figure 7S2A-B  DapRNAi  mCh  Dap^FL^  Dap^ΔD^  Dap^ΔCD^ | C155-GAL4, UAS-Dcr2/Y; UAS Dap160-RNAi/+  C155-GAL4/+; UAS-CD8::RFP/+  X=UAS-Dap^FL^::mCherry/+  X=UAS-Dap^ΔD^/+  X=UAS-Dap^ΔCD^/+ | 24 NMJ/6 larvae  22 NMJ/6 larvae  21 NMJ/6 larvae  16 NMJ/5 larvae  23 NMJ/6 larvae | ANOVA+Tukey’s multiple comparison test |
|  |  |  |  |
| 7S3A-C  Control  *nwk*^1/2^ | C155-GAL4/Y; UAS-GFP/+  C155-GAL4/Y; UAS-GFP/+; *nwk*^1^/*nwk*^2^*h* | 15 NMJ/4 larvae  19 NMJ/5 larvae | Mann-Whitney (B)  t-test (C) |
| Figure 7S3D-F  Dap^FL^  Dap^ΔD^  Dap^ΔCD^ | rescues= C155-GAL4/+; dap160^Δ1^/Df3450; X  X=UAS-Dap^FL^::mCherry/+  X=UAS-Dap^ΔD^/+  X=UAS-Dap^ΔCD^/+ | 16 NMJ/4 larvae  16 NMJ/4 larvae  16 NMJ/5 larvae | ANOVA+Tukey’s multiple comparison test |
